# Effects of sex ratio on adult fecundity, longevity and egg hatchability of *Bradysia difformis* Frey at different temperatures

**DOI:** 10.1371/journal.pone.0217867

**Published:** 2019-06-05

**Authors:** Yuping Gou, Guang Wang, Peter Quandahor, Qian Liu, Changzhong Liu

**Affiliations:** College of Plant Protection, Gansu Agricultural University, Biocontrol Engineering Laboratory of Crop Diseases and Pests, Lanzhou, Gansu Province, China; Chinese Academy of Agricultural Sciences Institute of Plant Protection, CHINA

## Abstract

The fungus gnat *Bradysia difformis* Frey not only attacks edible and medicinal fungi, it also causes the damages to leek, green onion, garlic and other vegetable crops. To confirm the effects of temperature and sex ratio on adult fecundity, longevity and egg hatchability of *B*. *difformis*, we assayed the adults survival longevity and numbers of oviposition, as well as egg hatching rate under female-male ratios of 1:1, 1:2, 1:3, 2:1 and 3:1 at 10, 15, 20, 25 and 30°C. Female fecundity and egg hatchability were increased with temperature and peaked at 25°C, which, however, were adverse to adult longevity. Furthermore, female-male ratio of 1:1, 1:2 and 1:3 seemed suitable for female fecundity, of which the sex ratio of 1:1 was the most suitable ratio for its oviposition behavior. These results provide an insight for predicting the population density of *B*. *difformis* and offer a literature reference in the effective treatment of harmful insects by controlling and changing the sex ratio artificially.

## Introduction

*Bradysia difformis* Frey (Diptera, Nematocera, Sciaridae and Bradysia) is a newly recorded species in mushroom greenhouses of China and becomes one of dominant pests causing heavy losses in edible and medicinal fungi [1–4], ornamental and flowering plants [5–7]. Its larvae are commonly known as fungus gnat, which drill into the mycelium, nearest substrates or sporocarps of fungus. In severe cases, the commercial value of protected environment such as greenhouses or mushroom cultures may be completely lost. According to the investigation from 2012 to 2013, *B*. *difformis*, not only attacks edible fungi, but also causes damages to leek, green onion, garlic and other vegetable crops [8]. It has become one of the main pests in leek growing areas of Tianshui, Gansu, China [8], and often occurs with *B*. *odoriphaga* Yang et Zhang [9].

Insects are ectotherm animals, so temperature is a particularly crucial to its growth, development, reproduction and behavior [10–16]. Temperature may directly exert an important influence on the metabolism of insects, and its metabolic rate varies with the temperature [12,15].

Sex ratio is an important characteristic of hermaphroditic organisms [17,18] and affected by internal genetics and external environment [19]. The 1:1 sex ratio is genetically stable because it ensures the parents make an equal genetic investment between females and males of the offspring [20]. However, there is a significant difference of sex ratio among dioecious organisms because of biological diversity in nature [21–23], leading to a distinct female or male dominance of the population. Zhang et al. (2008) conducted a study on the biological characteristics of *B*. *difformis* in the mushroom tunnels, and found that there were more females than males so that it was difficult to collect males, and the ratio of female to male was generally greater than 1:1 [1]. Different sex ratio structure will affect the mating and spawning of insect [24].

*Bradysia difformis* has become one of the most devasting pests of edible fungus, vegetable crops and flower plants, causing severe economic losses in China. Therefore, understanding how to efficiently control *B*. *difformis* is an urgent issue. The fecundity, longevity of adults and egg hatching rate under five sex ratio (1:1, 1:2, 1:3, 2:1 and 3:1) at five given temperatures (10, 15, 20, 25 and 30°C) are described and illustrated in this study. Realizing the influence of different temperature and sex ratio on female fecundity can predict the population density and the external factors that affect sex ratio. Our study also provides an important literature reference in the integrated pest management and effective treatment of harmful insects by controlling and changing the sex ratio artificially.

## Materials and methods

### Insect collection

Population of *Bradysia difformis* was collected from chives in the greenhouses of Tianshui (34°34’34”N, 105°42’32”), Gansu province of China. Two methods were used for its collection. One was that adults were collected with a trematode apparatus from the chive fields and placed in a transparent plastic pot covered with a wet filter paper for spawning. Another was that excavating the chives back to the laboratory, then put chive roots into a pot with gauze to collect adults, or singled out the larvae nearest the chive roots using a camel hair brush. Larvae were reared with chive rhizomes in moisturized petri dishes and the adults were reared in moisturized transparent plastic pots using filter paper [25].

### Host plant

Chives were used as diets of *B*. *difformis* and planted without using any pesticides in the outdoor experimental plots at Gansu Agricultural University (36°5’20”N, 103°41’54”E), Gansu province of China.

### Experiment conditions

Experiment was carried out in the intelligent artificial climate chamber (RXZ) at 10, 15, 20, 25 and 30°C five constant temperatures (exist 1°C errors), the larvae were under dark conditions, adults were under weak light (125 Lux, RH 80±5%). Each temperature was set for each of five female-male ratios of 1:1, 1:2, 1:3, 2:1 and 3:1.

### Culture method

Placed egg masses in 9-cm-diameter petri dishes containing a filter paper with suitable moisture. Larvae were fed with 2-3-mm chive rhizomes. The dishes were placed in the growth chamber at the experimental conditions set as mentioned above. Once the pupa appearance, it was placed in a single container. On the day when *B*. *difformis* adults emergence, males and females were paired according to the above ratios and subsequently kept in an individual rearing container (adult did not feed, while the filter paper should be kept moist but no excess water to ensure the spawning quality), with one pair per container. A total of 30 females were collected for study with each sex ratio. If the male died but female still survived, another male was supplied to meet the need for female fecundity. The fecundity and longevity of adults were observed everyday 2 times until all adults had died. The spawned eggs were raised in the same environment as the adults, the number of hatching eggs were then recorded and the hatching rates were calculated.

### Statistical analysis

Excel 2007 and SPSS 19.0 were used for data statistics analysis. One-way analysis of variance was used to compare adult, fecundity, longevity and egg hatch-ability (Tukey, *P < 0*.*05*). Linear regression analysis was used to illustrate relationships between sex ratio and egg hatching rate at different temperatures. No data transformation was carried out.

## Results

### Female fecundity

**Fig 1:**
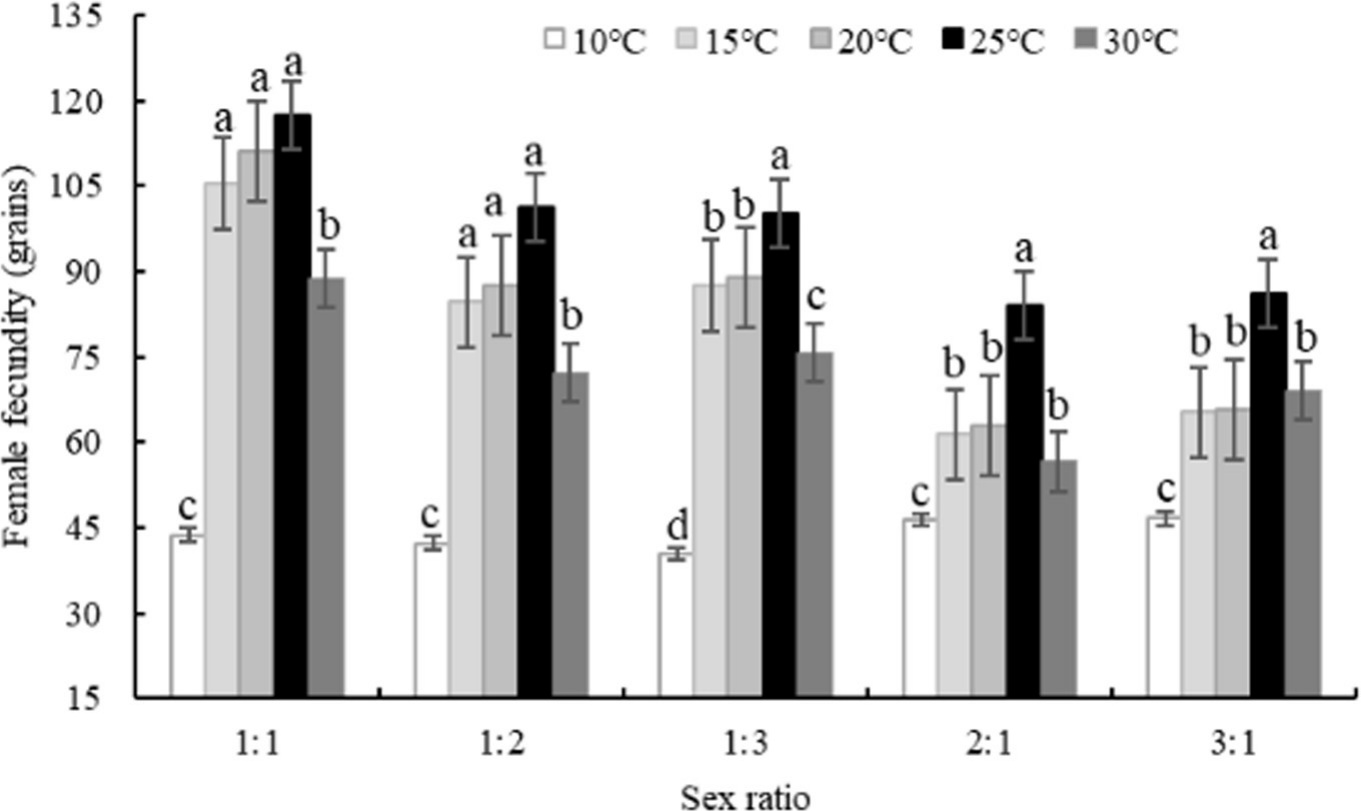
Effect of sex ratio on female fecundity of *Bradysia difformis* at five temperatures(Mean± SE; Different small letters of the same sex ratio represent significant difference among five temperatures (P < 0.05). The same to the following figures.).

The female fecundity of *Bradysia difformis* with different sex ratios at five temperatures are plotted in Fig 1. Different temperatures at the same sex ratio had remarkable effects on female fecundity (*P < 0*.*05*). When female-male ratio was 1:1, the fecundity was significantly greater (*P < 0*.*05*) at 25°C (117.30 grains), 20°C (111.05 grains) and 15°C (105.43 grains) than other temperatures (10 and 30°C), of which at 10°C the fecundity became the lowest (43.70 grains). When the female-male ratio was 1:2, there was a significant difference (*P < 0*.*05*) in the female fecundity between 25°C (101.20 grains) and 10°C (42.20 grains). In addition, when under ratios of 1:3, 2:1, and 3:1, the fecundity was 100.20, 83.95 and 86.20 grains at the temperatures of 25°C, which was higher than those of 10, 15, 20 and 30°C temperatures.

Different female-male ratios at the same temperature had little influences on the female fecundity of *B*. *difformis* (Fig 1). Under temperature of 10°C, oviposition was the lowest among five sex ratios, especially at the ratio of 1:3 (40.4 grains), which was significantly different (*P < 0*.*05*) with the ratios of 1:1 (43.7 grains), 1:2 (42.2 grains), 2:1 (46.35 grains) and 3:1 (46.63 grains). Under temperatures of 15 and 20°C, oviposition was higher at the ratios of 1:1, 1:2 than 1:3, 2:1, 3:1 and had significant difference (*P < 0*.*05*). Under temperatures of 25°C, oviposition was the highest among five sex ratios, especially at the ratio of 1:1 (117.3 grains). Under temperatures of 30°C, oviposition had no significant difference among ratios of 1:1, 1:2, 1:3, 2:1 and 3:1, which were 88.7, 72.2, 75.7, 56.65 and 69 grains, respectively.

In short, female fecundity of *B*. *difformis* was higher at the sex ratio of 1:1 than ratios of 1:2, 1:3, 2:1 and 3:1 under temperatures of 10, 15, 20, 25 and 30°C.

### Egg hatching rate

**Fig 2:**
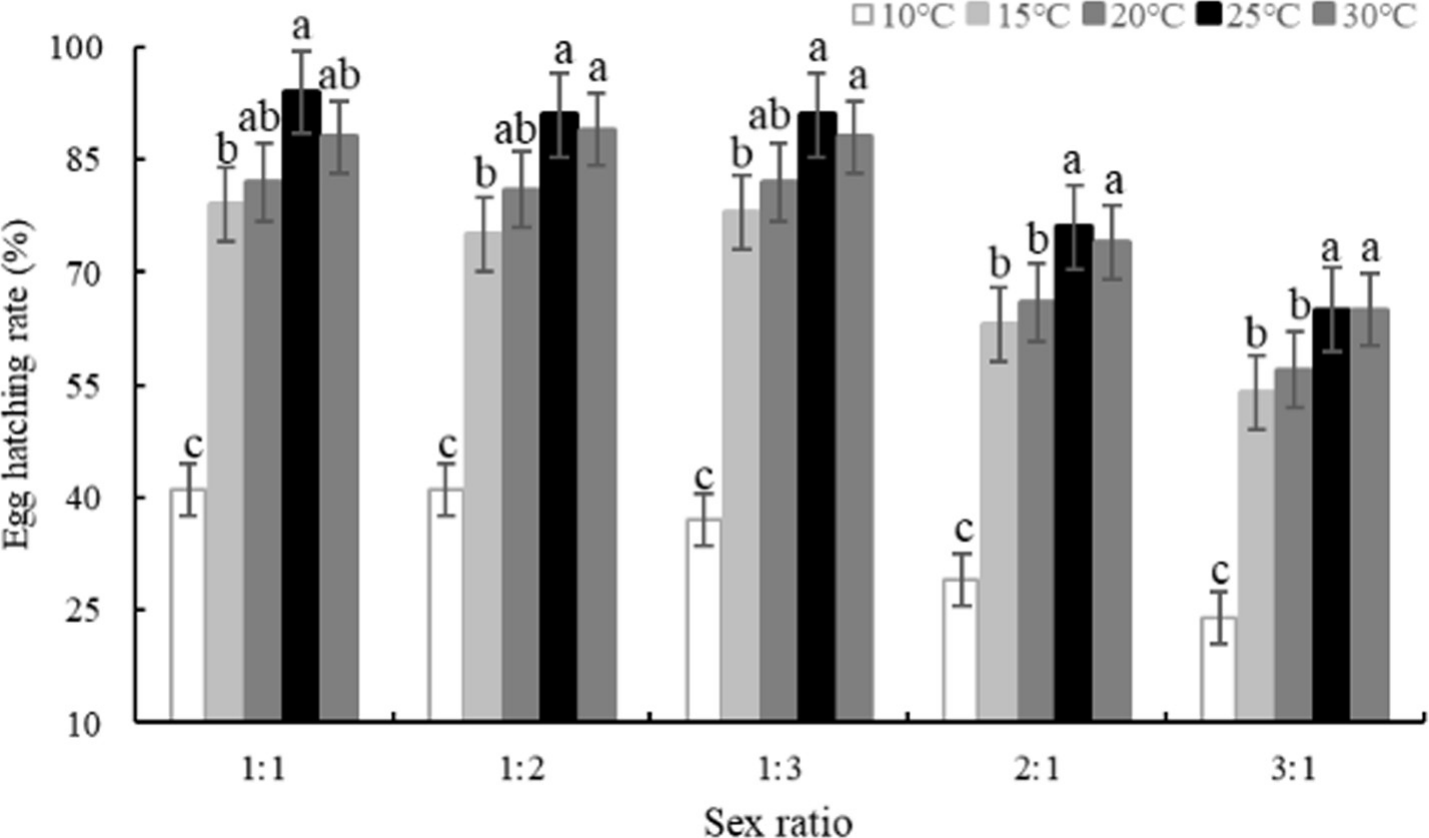
Effect of sex ratio on the egg hatching rate of *Bradysia difformis* at five temperatures.

With female-male ratios of 1:1, 1:2 and 1:3, the egg hatching rate of *B*. *difformis* was higher (*P < 0*.*05*) at 20 to 30°C (Fig 2) and ranged from 81.33 to 93.67%. With 2:1 sex ratio, the hatching rate was intended to be greater at 25 and 30°C than at other temperatures and were 29, 63, 66, 76 and 74%, respectively from 10 to 30°C. The lowest hatching rate was recorded at 10°C for an average of 34.40% among female-male ratios of 1:1, 1:2, 1:3, 2:1 and 3:1. The egg hatching rate was increased gradually with the increasing temperature from 10 to 25°C, but began to decrease at 30°C with a maximum average of 83.40% at 25°C under all five sex ratios.

### Relationships between sex ratio and egg hatching rate

**Fig 3:**
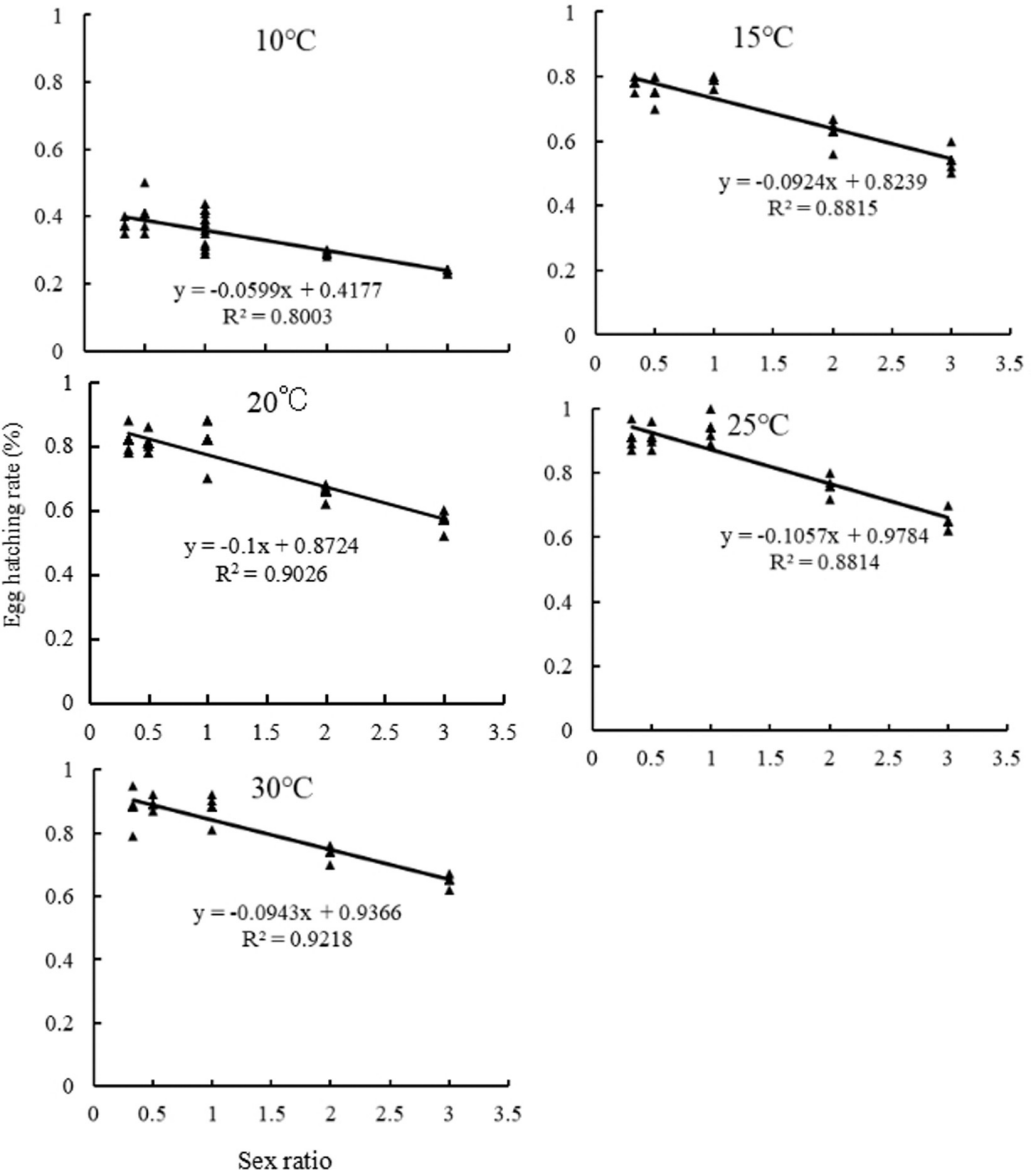
Relationships between sex ratio and egg hatching rate of *Bradysia difformis* at five temperatures.

In order to illustrate the effect of temperature and sex ratio on egg hatching rate of *B*. *difformis* more accurately and intuitively, we assayed the relationships between sex ratio and egg hatching rate at the temperatures of 10, 15, 20, 25 and 30°C separately by using Linear Regression (Fig 3). The results showed that there was a negative correlation between the egg hatching rate and the female-male ratio (*P<0*.*05*), showing that the egg hatching rate was decreased gradually with the increasing of female-male ratios.

### Adult longevity

**Fig 4:**
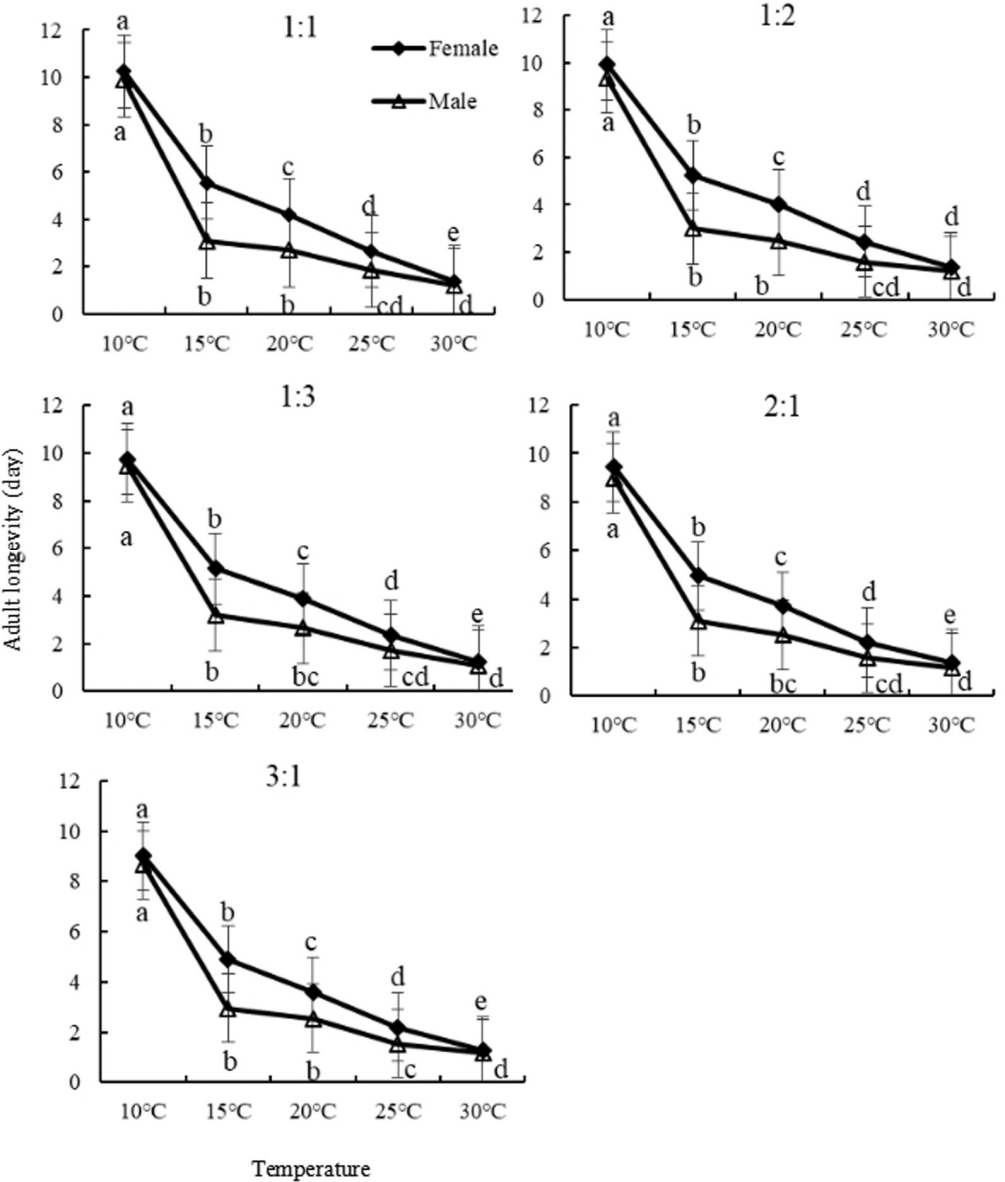
Female and male longevity of *Bradysia difformis* at different sex ratios at five temperatures.

Adult longevity at the temperatures of 10, 15, 20, 25, 30°C and the female- male ratios of 1:1, 1:2, 1:3, 2:1, 3:1 were determined in this study. We found that temperature had significant effects (*P<0*.*05*) on male and female longevity, which was decreased with the increasing of temperature at five sex ratios. Male and female longevity was the longest at 10°C averaged of 9.90 d for male and 10.25 d for female, whereas the shortest at 30°C averaged 1.10 d for male and 1.25 d for female (Fig 4), indicating the longevity became shorter with increasing temperature. Sex ratio had little influences on adults longevity under the same temperature. However, their longevity was longer at 1:1 sex ratio among five temperatures rather than 3:1.

## Discussion

Fecundity, life history and fitness of insects are greatly affected by temperature because of ectothermic animal [26]. The effect of temperature on insect fecundity is mainly in its adult stage [27]. Although the adult can survive, or have more longevity as well as larger individual body at lower temperature, it will lead to point where male and female can’t finish mating smoothly or reproduce rarely because of their immature sex gland [27]. Adult lifespan of insects is shorter at higher temperature, additionally, the sperm of male is difficult to form or have lost its active ability under such environmental conditions. As a result, it affects the mating behavior and causes sterility, consequently affects the hatchability of eggs [28]. Mating and spawning ability are closely related to their female-male ratio structure, which influence the population growth of insect. Under a certain sex ratio, it is obviously known that higher the fecundity of adult is, faster the population growth is [29].

The present results demonstrate that temperature has significant effects on the behaviors of *B*. *difformis* like fecundity, longevity and hatchability. Furthermore, those behaviors of reproduction and hatchability are more active ranging from 15 to 25°C and it could not reproduce at 30°C. However, longevity shortened with increasing temperature and stayed a longer time at 10°C. The wide scope of temperatures at which *B*. *difformis* can survive and reproduce helps explain why its management is pretty difficult.

Previous studies have shown that lower or higher sex ratio will hinder the mating and reproductive ability of both male and female adult insects, and thus adversely affect the reproduction of insect population [30–31]. The results of this experiment also confirmed that whether the female fecundity of *B*. *difformis* increased or decreased was closely related to the female-male ratio. It can be seen that fecundity of *B*. *difformis* is obviously affected by the sex ratio of the adult. In addition, the egg number of female spawn varies with the sex ratio but it can keep higher fecundity under a certain sex ratio. The female-male ratio leads to the decrease of female fecundity, which may be due to the competition with high population density in a certain space, resulting in the interference with the mating activity. This phenomenon indicates that it is possible to use insect sex attractants to trap the males and destroy the sex ratio which can be applied for effectively controlling *B*. *difformis*.

The hatching rate of *B*. *difformis* at higher male ratio is significantly greater than that of the high female ratio, which may be due to more unfertilized eggs were spawned by un-mated females thus unable to hatch.

## Conclusions

Our present study has provided fundamental information on the effects of sex ratios on the adult fecundity, longevity and egg hatchability of *B*. *difformis* at different temperatures. Although our data are slightly different because of controlled environment from the natural population with much unknown factors, it can provide an insight for predicting the population density of *B*. *difformis* and offer a reference literature in the effective treatment of harmful insects by controlling and changing the sex ratio artificially.
